# Proteomic analysis indicates massive changes in metabolism prior to the inhibition of growth and photosynthesis of grapevine (*Vitis vinifera* L.) in response to water deficit

**DOI:** 10.1186/1471-2229-13-49

**Published:** 2013-03-21

**Authors:** Grant R Cramer, Steve C Van Sluyter, Daniel W Hopper, Dana Pascovici, Tim Keighley, Paul A Haynes

**Affiliations:** 1 Department of Biochemistry and Molecular Biology, University of Nevada Reno, Reno, NV 89557, USA; 2 Present address: Department of Chemistry and Biomolecular Sciences, Macquarie University, North Ryde, NSW 2109, Australia

## Abstract

**Background:**

Cabernet Sauvignon grapevines were exposed to a progressive, increasing water defict over 16 days. Shoot elongation and photosynthesis were measured for physiological responses to water deficit. The effect of water deficit over time on the abundance of individual proteins in growing shoot tips (including four immature leaves) was analyzed using nanoflow liquid chromatography - tandem mass spectrometry (nanoLC-MS/MS).

**Results:**

Water deficit progressively decreased shoot elongation, stomatal conductance and photosynthesis after Day 4; 2277 proteins were identified by shotgun proteomics with an average CV of 9% for the protein abundance of all proteins. There were 472 out of 942 (50%) proteins found in all samples that were significantly affected by water deficit. The 472 proteins were clustered into four groups: increased and decreased abundance of early- and late-responding protein profiles. Vines sensed the water deficit early, appearing to acclimate to stress, because the abundance of many proteins changed before decreases in shoot elongation, stomatal conductance and photosynthesis. Predominant functional categories of the early-responding proteins included photosynthesis, glycolysis, translation, antioxidant defense and growth-related categories (steroid metabolism and water transport), whereas additional proteins for late-responding proteins were largely involved with transport, photorespiration, antioxidants, amino acid and carbohydrate metabolism.

**Conclusions:**

Proteomic responses to water deficit were dynamic with early, significant changes in abundance of proteins involved in translation, energy, antioxidant defense and steroid metabolism. The abundance of these proteins changed prior to any detectable decreases in shoot elongation, stomatal conductance or photosynthesis. Many of these early-responding proteins are known to be regulated by post-transcriptional modifications such as phosphorylation. The proteomics analysis indicates massive and substantial changes in plant metabolism that appear to funnel carbon and energy into antioxidant defenses in the very early stages of plant response to water deficit before any significant injury.

## Background

Technical and analytical advances over the last decade have dramatically changed our capabilities for gene discovery and functional genomics. Today there are many more transcriptomics analyses published than proteomics analyses of plants [1]. Metabolites are the direct product of enzymatic reactions and the correlation of transcriptomics and proteomics profiles are generally 50% or lower, emphasizing the need for a greater focus on proteomics to fully understand plant growth and metabolism and their responses to environmental stress.

Ion toxicity and water deficit contribute to the inhibition of growth of most salt-stressed plants [2-4]. Osmotic shock experiments are typically performed to separate osmotic from ion toxicity effects [2,3]. This type of experiment can provide useful information on the initial responses to sudden and severe stress [5-7], however, a gradual application of stress reveals a more complex plant response to the stress [5].

In an earlier study [8], we investigated the gradual effects of water deficit and salinity on the proteome of two grapevine cultivars, Chardonnay and Cabernet Sauvignon. Three time points (0, 8 and 16 days) were examined at the protein level only. Distinct differences in response to the stresses were found between the cultivars, but the time course lacked the resolution to discern early events in the responses of the vines to stress.

In order to gain deeper insights into the response of grapevine to water deficit and salinity, a larger number of time points were collected from a single cultivar, and transcript and metabolite profiles were assayed from aliquots of the same samples. Water deficit and salinity stress were applied gradually over a 16-day-period to pot-grown Cabernet Sauvignon vines in the greenhouse [9]. Water was withheld over the course of the experiment for some plants and salt (NaCl:CaCl_2_ equal to 10:1) was applied gradually to other vines to effectively mimic the drop in stem water potential in the water-deficit-stressed vines. There were large and distinct differences in steady-state transcript abundance as measured by microarray analysis among the control, water-deficit and salt-stressed vines [9]. Stress lowered stem water potentials equally in the stressed vines, but the growth of water-deficit-stressed vines was reduced more severely than salt-stressed vines. The inhibition of shoot elongation was detected 4 days after application of the stress treatments even though significant differences in stem water potentials were not detectable. The first significant differences in transcript abundance due to stress were detectable after 8 days of stress treatment when water potential began to decline significantly. Significant differences were found between the stress treatments in both the timing and the abundance of transcripts affected by the stress. Metabolite profiles of sugars, organic acids and amino acids were linked to transcript profiles revealing that photosynthesis, gluconeogenesis and photorespiration were affected by the stress treatments. Water-deficit-treated vines appeared to have greater demand for osmotic adjustment and detoxification of free radicals produced by photoinhibition than salt-stressed vines.

In the present proteomics study, a similar water deficit experiment was repeated to get additional details on the physiological and metabolic responses of Cabernet Sauvignon vines. One striking discovery from this analysis was that there were massive changes in protein abundance in a number of metabolic pathways prior to the inhibition of growth and photosynthesis. The early changes in protein abundance (e.g. increase in photosynthetic proteins and decrease in growth-related proteins) appear to be an acclimation response in anticipation of an upcoming water deficit. The later changes in protein abundance appear to be largely in response to significant stress injury (e.g. photorespiration).

## Results

The water deficit experiment was repeated in a similar manner as before [9] with 2-year-old Cabernet Sauvignon grapevines grown in pots in a greenhouse under nearly identical conditions. Control vines were watered daily with tap water, whereas water-deficit-treated vines were not irrigated. Shoot elongation, photosynthesis, leaf conductance and pot weight were monitored over the course of the 16-day experiment to determine how the stress progressed.

**Figure 1:**
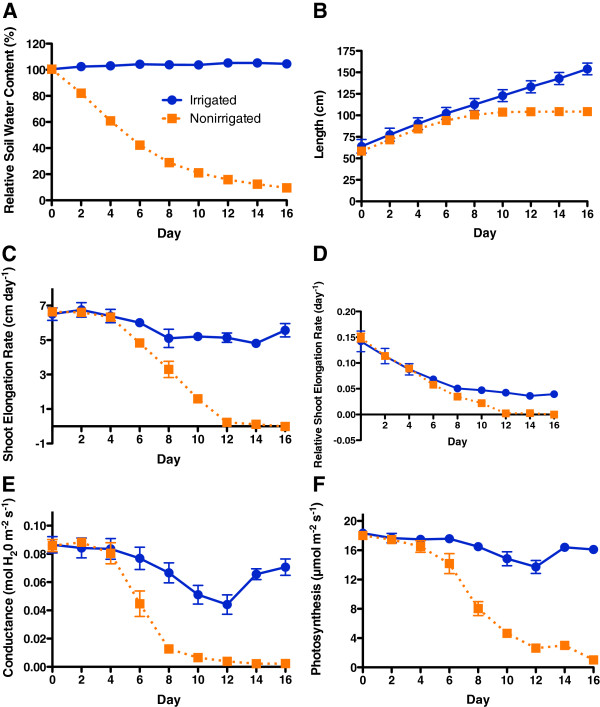
**Physiological responses of Cabernet Sauvignon grapevines to water deficit.** A time course of measurements of **A**) pot relative water content, **B**) shoot elongation, **C**) shoot elongation rate, **D**) relative shoot elongation rate, **E**) photosynthesis and **F**) stomatal conductance of grapevines in control (closed blue circles, solid line) and water deficit treatment (closed orange squares, dotted line). Data are the means ± SE; n = 5.

Pot weight declined immediately after the start of the experiment and reached about 10% relative water content by Day 16 (Figure 1A). Vine growth, stomatal conductance and photosynthesis declined significantly by Day 6 for nonirrigated vines (Figure 1B, C, D, E, and F). Shoot elongation was measured every two days, therefore the decline at Day 6 actually began sometime between Day 4 and 6. Shoot elongation rate (Figure 1C) was a more sensitive indicator of stress, making it easier to visualize the stress effects than either shoot length (Figure 1B) or relative shoot elongation rate (Figure 1D). Photosynthesis measurements were measured over a 5-minute period, and were essentially instantaneous at the moment of measurement. Shoot elongation had stopped and stomata were nearly closed by Day 12 (Figure 1C and E). Photosynthesis was inhibited progressively with time after Day 4 in the nonirrigated vines (Figure 1F).

Proteins were extracted from the growing shoot tips (apex, stem, tendrils and four immature leaves) with a modified phenol-based protocol that is regularly used in the Cramer lab [10] and digested with trypsin in preparation for peptide analysis using nanoflow liquid chromatography-mass spectrometry (nanoLC-MS/MS). Peptide spectra were analyzed and quantified using both open source and custom-based bioinformatics software (see Methods for details). Protein identifications were filtered and false discovery rates (FDRs) calculated. Protein quantity was estimated as normalized spectral abundance factors (NSAF; see Methods). Approximately 27,000 spectra per sample were assigned to peptides matching 2277 *Vitis vinifera* proteins in the UniProtKB database (Additional file 1). The average CV for all 2277 proteins was approximately 9%.

Gene ontology (GO) categories of the identified proteins were downloaded from the UniProt database (uniprot.org) and assessed in BinGO (Additional file 2). There were hundreds of categories (850) with 240 significantly over-represented GO categories compared to the whole proteome at UniProt after FDR correction. Thus, there was wide representation of different classes of proteins in this dataset. The major categories included protein metabolic process, translation, organic acid metabolism, sugar metabolism, photosynthesis and protein folding.

The proteins were placed into absent (0) or present (1) categories for each treatment (Additional file 1). If any treatment had a protein missing in at least one of its replicates it was designated as absent. For example, 942 of the 2277 proteins were present in all replicates in all treatments (Additional file 1, key = 11111111 for Control Day 4, 8, 12, 16, Water Deficit Day 4, 8, 12, 16, respectively). Twenty-three proteins were present in the control vines, but missing altogether from the nonirrigated vines in at least one of the replicates for each day of the water deficit treatment (key = 11110000); another 125 proteins were missing in nonirrigated vines on Day 16 (key = 11111110) and 69 proteins were missing in nonirrigated vines on Day 12 and 16 (key = 11111100). Another interesting group of 20 proteins was consistently present on Day 12 and 16 of the water deficit treatment (key = 00000011). Some of these proteins may be missing because their average abundance was near the threshold for detection and were simply missing from a sample due to random variation. In other cases (e.g. key = 11111110; 00000011), some of the proteins in this category had substantial spectral counts and then disappeared or appeared on the specific day (e.g. Day 12 or 16 of the water deficit treatment).

**Figure 2:**
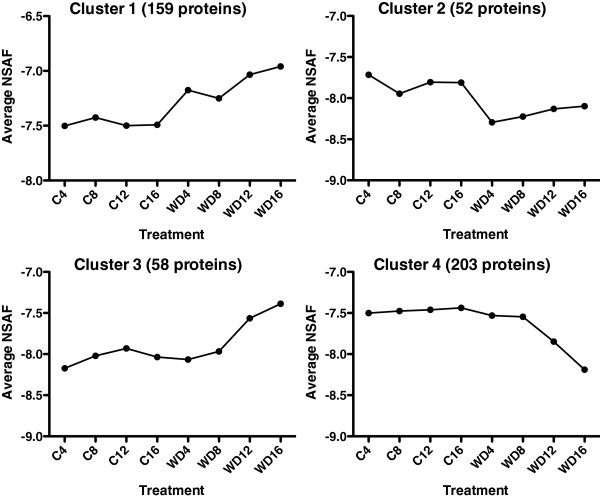
**Cluster analysis of the 942 proteins present in all shoot tip samples.** NSAF stands for normalized spectral abundance factors, a measure of relative protein abundance.

NSAF data were analyzed further for the 942 proteins present in all samples. Treatment effects and their interaction with time were analyzed by 2-way ANOVA with a FDR of 0.05 using the MultiExperiment Viewer (MeV) in the TM4 software package [11]. There were 431 and 241 proteins affected significantly by the stress treatment and the interaction term, respectively (Additional file 3). There were 200 proteins common to both Treatment Effect and Treatment x Day Interaction resulting in 472 unique proteins affected by water deficit out of the 942 proteins (50%). These 472 proteins affected by the water deficit were separated into four different clusters using the k-means/medians clustering algorithm in the MeV package (Figure 2).

Cluster 1 consisted of 159 proteins that increased significantly on Day 4 and remained elevated for the remainder of the water deficit treatment. A large number of GO biological process functional categories were overrepresented in this cluster (Additional file 4). The most significant category was photosynthesis based upon the p-value, followed by photorespiration and oxidation reduction. Other categories of interest included hexose metabolism, chromosome organization and oxidative stress (antioxidant) responses.

**Figure 3:**
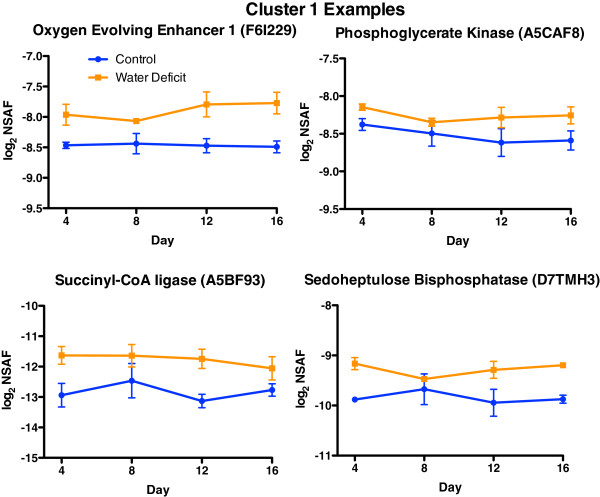
**The effect of water deficit on the relative abundance of representative proteins for Cluster 1.** Data are the means ± SE; n = 3.

Some representative examples of proteins of this cluster involving carbohydrate and energy metabolism (Figure 3) are oxygen evolving enhancer 1 (PSBO2), phosphoglycerate kinase (PGK1), succinyl-CoA ligase, and sedoheptulose bisphosphatase (SBPASE). The abundance of these proteins was increased significantly at Day 4 in nonirrigated vines and remained elevated throughout the stress treatment. PSBO2 stabilizes the catalytic Mn cluster of photosystem II and regulates the turnover of the D1 reaction center protein [12]. PGK1 catalyzes the reversible transfer of phosphate between 3-phosphoglycerate and ATP with 1,3 bisphosphoglycerate and ADP in the Calvin-Benson cycle (photosynthesis), glycolysis and gluconeogenesis [13]. Succinyl-CoA ligase converts succinyl-CoA and ADP to succinate and ATP in the citric acid cycle and SBPASE is involved in the regeneration phase of the Calvin-Benson cycle [13].

**Figure 4:**
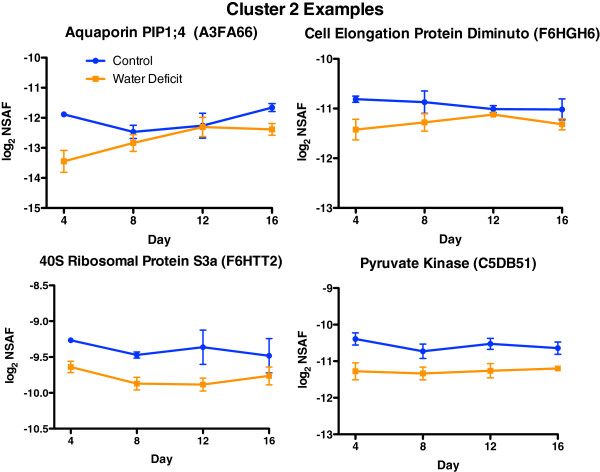
**The effect of water deficit on the relative abundance of representative proteins for Cluster 2.** Data are the means ± SE; n = 3.

Cluster 2 consisted of 52 proteins whose abundance immediately declined on Day 4 of the water deficit treatment. The major biological processes significantly over-represented in Cluster 2 included protein metabolism (e.g. translation, proteosomal, ribosomal and chaperone proteins, phosphorylation and deacetylation activities), and glycolysis (Additional file 5). Examples of proteins in this cluster (Figure 4) include a plasma membrane intrinsic protein (PIP1;4), Diminuto/DWF1, a 40S ribosomal protein (S3a) and pyruvate kinase (PK). PIP1;4 is a subclass of aquaporins that have low water transport properties in vitro, but may have interactive effects with PIP2 proteins which are known to transport water [14]. The expression of grapevine aquaporins is correlated with the hydraulic conductance of the root and growth of the shoot [15]. Diminuto/DWF1 is involved in brassinosteroid biosynthesis and plant growth [16]. Mutants of this gene have dwarf phenotypes. Applications of brassinosteroids to drought-stressed plants seem to improve plant responses to water deficit in some plants [17] and a recent proteomic study implicated brassinosteroid biosynthesis in a response of a resurrection plant to dehydration [18]. The 40S ribosomal protein S3a is a structural subunit of the ribosome involved in protein translation in the cytoplasm [19]. Pyruvate kinase catalyzes the conversion of phosphoenolpyruvate to pyruvate and is an important regulator of glycolysis [19,20].

**Figure 5:**
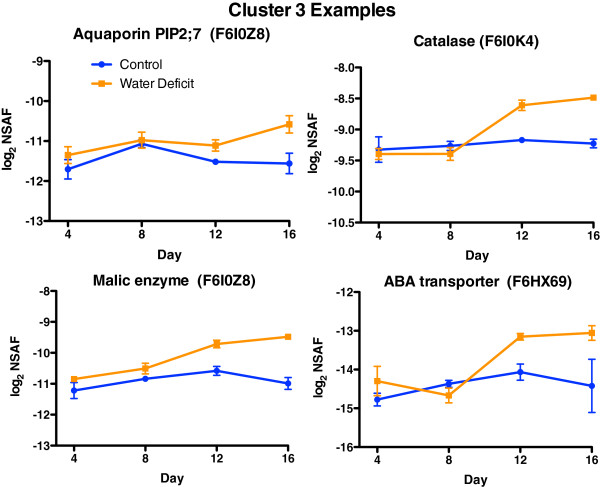
**The effect of water deficit on the relative abundance of representative proteins for Cluster 3.** Data are the means ± SE; n = 3.

The abundance of the 58 proteins in Cluster 3 progressively increased during the water deficit treatment (especially on Day 12 and 16). Many of these proteins are involved in stress responses including response to reactive oxygen species, carbohydrate metabolism (e.g. gluconeogenesis and glycolysis), and various transporters (Additional file 6). Representative examples of these proteins and their abundance profiles are shown in Figure 5 including a plasma membrane intrinsic protein (PIP2;7) that is involved in water transport, catalase that is involved in antioxidant defense [21], malic enzyme that is involved in malate metabolism [22] and an ABA transporter, whose *Arabidopsis* ortholog transports ABA across the plasma membrane into guard cells and other plant cell types [23]. Additional interesting proteins were identified in the present and absent category, 00000011, including a dehydrin, a calcium dependent protein kinase, a peroxidase and a nitrate/chloride transporter (Additional file 1).

Cluster 4 consisted of 203 proteins that progressively decreased in abundance during the stress and were primarily involved in protein metabolism (translation and folding) and amino acid metabolism (Additional file 7). However, there were many other processes affected, such as gene expression, carbohydrate and phenylpropanoid metabolism. Furthermore, 34 of the 125 missing proteins on Day 16 (key = 11111110) whose average spectral counts were substantially higher than zero matched this decreasing profile (see Additional File 1). Most of these proteins missing on Day 16 of the water deficit treatment fit into the general C metabolism category.

**Figure 6:**
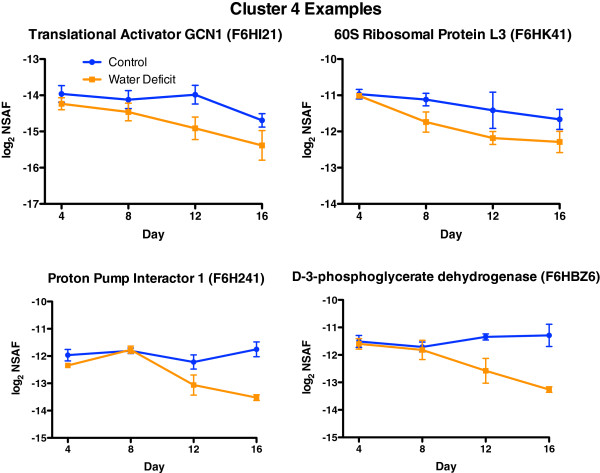
**The effect of water deficit on the relative abundance of representative proteins for Cluster 4.** Data are the means ± SE; n = 3.

Figure 6 displays four protein profile examples from Cluster 4. GCN1 (general control of amino acid biosynthesis) regulates the GCN2 kinase that regulates translation elongation during amino acid starvation [24]. The 60S ribosomal protein L3 is a subunit of the ribosome involved in translation. The proton pump interactor 1 regulates the plasma membrane proton pump [25] and 3-phosphoglycerate dehydrogenase is the first enzyme involved in the serine biosynthesis pathway [26].

**Figure 7:**
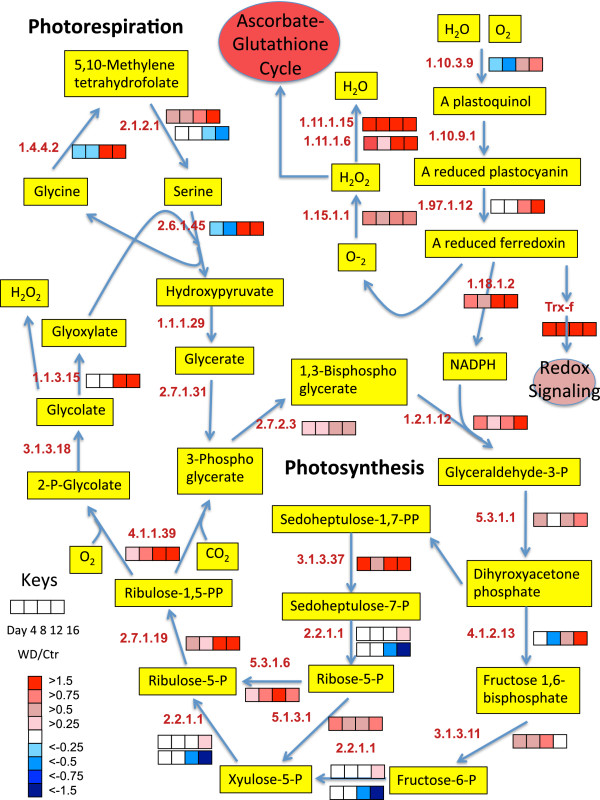
**The effect of water deficit on the photosynthesis and photorespiration pathways over time.** The boxes represent protein expression levels of the ratio of water deficit treatment (WD) to control treatment (Ctr) as defined in the key. Boxes from left to right are ratios for Days 4 through 12. EC numbers for the following proteins are: 1.1.1.29 (glycerate dehydrogenase), 1.1.3.15 (glycolate oxidase), 1.2.1.12 (glyceraldehyde-3-phosphate dehydrogenase), 1.4.4.2 (glycine dehydrogenase), 1.10.3.9 (photosystem II D2 protein), 1.10.9.1 (cytochrome b6-f complex iron-sulfur subunit), 1.11.1.6 (catalase), 1.11.1.15 (peroxiredoxin), 1.15.1.1 (superoxide dismutase), 1.18.1.2 (ferredoxin-NADP reductase), 1.97.1.12 (photosystem I P700 chlorophyll a apoprotein), 2.1.2.1 (serine hydroxymethyltransferase), 2.2.1.1 (transketolase), 2.6.1.45 (serine-glyoxylate aminotransferase), 2.7.1.19 (phosphoribulokinase), 2.7.1.31 (glycerate kinase), 2.7.2.3 (phosphoglycerate kinase), 3.1.3.11 (fructose-1,6-bisphosphatase), 3.1.3.18 (phosphoglycolate phosphatase), 3.1.3.37 (sedoheptulose-1,7-bisphosphatase), 4.1.1.39 (RuBisCo large subunit), 4.1.2.13 (fructose-bisphosphate aldolase), 5.1.3.1 (ribulose-phosphate 3-epimerase), 5.3.1.1 (triosephosphate isomerase), 5.3.1.6 (ribose 5-phosphate isomerase), Trx-f (thioredoxin-f).

**Figure 8:**
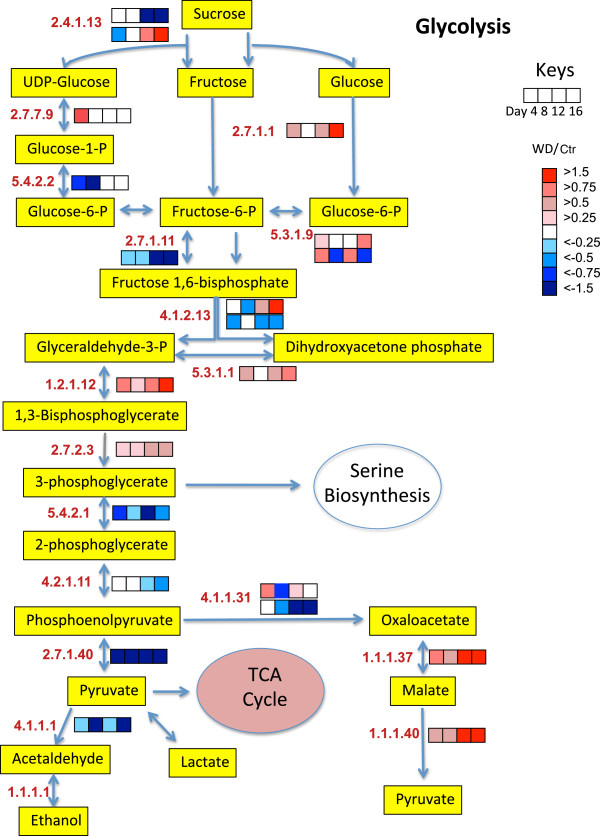
**The effect of water deficit on glycolysis over time.** The boxes represent protein expression levels of the ratio of water deficit treatment (WD) to control treatment (Ctr) as defined in the key. Boxes from left to right are ratios for Days 4 through 12. EC numbers for the following proteins are: 1.1.1.1 (alcohol dehydrogenase ), 1.1.1.37 (malate dehydrogenase), 1.1.1.40 (malic enzyme), 1.2.1.12 (glyceraldehyde-3-phosphate dehydrogenase), 2.4.1.13 (sucrose synthase), 2.7.1.1 (hexokinase-1), 2.7.1.11 (phosphofructokinase), 2.7.1.40 (pyruvate kinase), 2.7.2.3 (phosphoglycerate kinase), 2.7.7.9 (UTP-glucose-1-phosphate uridylyltransferase) 4.1.1.1 (pyruvate decarboxylase), 4.1.1.31 (phosphoenolpyruvate carboxylase), 4.1.2.13 (fructose-bisphosphate aldolase), 4.2.1.11 (enolase), 5.3.1.9 (glucose-6-phosphate isomerase), 5.4.2.1 (phosphoglycerate mutase), 5.4.2.2 (phoshoglucomutase), 5.3.1.1 (triose phosphate isomerase).

**Figure 9:**
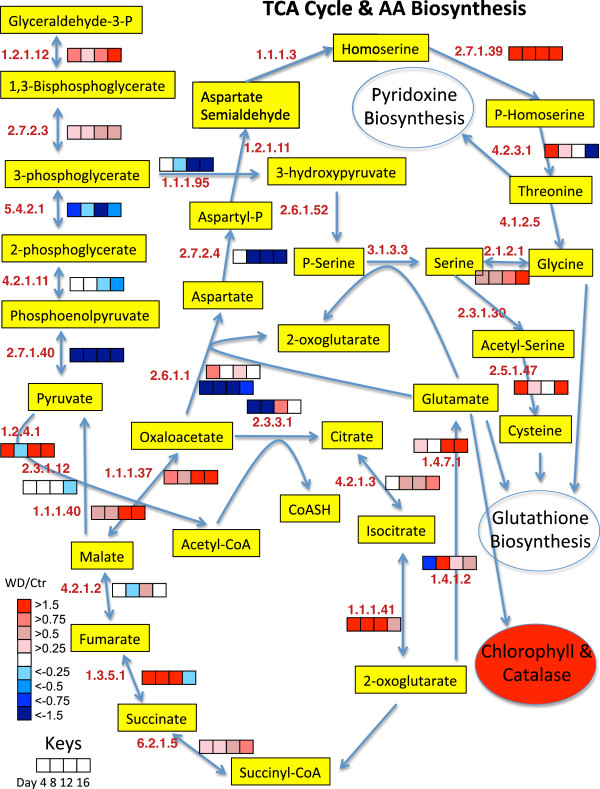
**The effect of water deficit on the TCA cycle, glutamate, glycine, serine, threonine and cysteine biosynthesis pathways over time.** The boxes represent protein expression levels of the ratio of water deficit treatment (WD) to control treatment (Ctr) as defined in the key. Boxes from left to right are ratios for Days 4 through 12. EC numbers for the following proteins are: 1.1.1.3 (homoserine dehydrogenase), 1.1.1.37 (malate dehydrogenase), 1.1.1.40 (malic enzyme), 1.1.1.41 (isocitrate dehydrogenase), 1.1.1.95 (D-3-phosphoglycerate dehydrogenase), 1.2.1.11 (aspartate-semialdehyde dehydrogenase), 1.2.1.12 (glyceraldehyde-3-phosphate dehydrogenase), 1.2.4.1 (pyruvate dehydrogenase E1 component subunit alpha), 1.3.5.1 (succinate dehydrogenase), 1.4.1.2 (glutamate dehydrogenase), 1.4.7.1 (ferredoxin-dependent glutamate synthase), 2.1.2.1 (serine hydroxymethyltransferase), 2.3.1.30 (serine O-acetyltransferase), 2.3.3.1 (citrate synthase), 2.5.1.47 (cysteine synthase), 2.6.1.1 (aspartate aminotransferase), 2.6.1.52 (phosphoserine aminotransferase), 2.7.1.39 (homoserine kinase), 2.7.1.40 (pyruvate kinase), 2.7.2.3 (phosphoglycerate kinase), 2.7.2.4 (aspartate kinase-homoserine dehydrogenase), 3.1.3.3 (phosphoserine phosphatase), 4.1.2.5 (threonine aldolase), 4.2.1.2 (fumarate hydratase), 4.2.1.3 (aconitate hydratase), 4.2.1.11 (enolase), 4.2.3.1 (threonine synthase), 5.4.2.1 (phosphoglycerate mutase), 6.2.1.5 (succinyl-CoA ligase).

**Figure 10:**
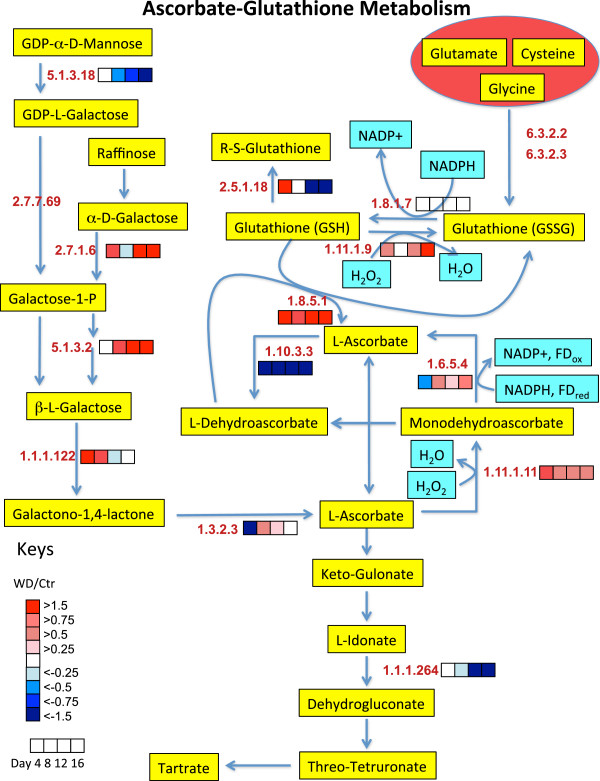
**The effect of water deficit on ascorbate-glutathione metabolism over time.** The boxes represent protein expression levels of the ratio of water deficit treatment (WD) to control treatment (Ctr) as defined in the key. Boxes from left to right are ratios for Days 4 through 12. EC numbers for the following proteins are: 1.1.1.122 (D-arabinose 1-dehydrogenase), 1.1.1.264 (L-idonate 5-dehydrogenase), 1.3.2.3 (L-galactono-1,4-lactone dehydrogenase), 1.6.5.4 (monodehydroascorbate reductase), 1.8.1.7 (glutathione reductase), 1.8.5.1 (dehydroascorbic reductase), 1.10.3.3 (L-ascorbate oxidase), 1.11.1.9 (glutathione peroxidase), 1.11.1.11 (L-ascorbate peroxidase), 2.5.1.18 (glutathione S-transferase PARB), 2.7.1.6 (galactokinase), 2.7.7.69 (GDP-L-galactose phosphorylase), 5.1.3.2 (UDP-glucose 4-epimerase), 5.1.3.18 (GDP-mannose 3,5-epimerase), 6.3.2.2 (glutamate--cysteine ligase), 6.3.2.3 (glutathione synthase).

**Figure 11:**
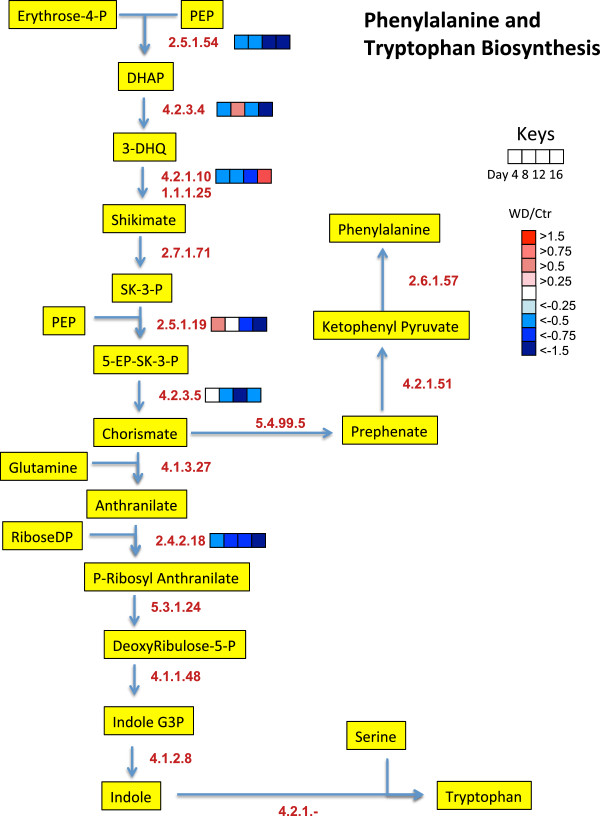
**The effect of water deficit on the phenylalanine and tryptophan biosynthesis pathways over time.** The boxes represent protein expression levels of the ratio of water deficit treatment (WD) to control treatment (Ctr) as defined in the key. Boxes from left to right are ratios for Days 4 through 12. EC numbers for the following proteins are: 2.4.2.18 (anthranilate phosphoribosyltransferase), 2.5.1.19 (3-phosphoshikimate 1-carboxyvinyltransferase), 2.5.1.54 (Phospho-2-dehydro-3-deoxyheptonate aldolase), 2.6.1.57 (aromatic-amino-acid transaminase), 2.7.1.71 (shikimate kinase), 4.1.1.48 (indole-3-glycerol phosphate synthase), 4.1.2.8 (indole-3-glycerol-phosphate lyase), 4.1.3.27 (anthranilate synthase), 4.2.1.51 (prephenate dehydratase), 4.2.1.122 (tryptophan synthase), 4.2.3.4 (3-dehydroquinate synthase), 4.2.3.5 (chorismate synthase), 4.2.1.10 1.1.1.25 (3-dehydroquinate dehydratase / shikimate dehydrogenase), 5.3.1.24 (phosphoribosylanthranilate isomerase), 5.4.99.5 (chorismate mutase).

To get a better understanding of the biochemical processes in the growing shoot tips of grapevines affected by water deficit over time, a large number of detected proteins were displayed in newly constructed metabolic maps that were based upon previously generated maps in AraCyc [27], KEGG [28], and VitisNet [29]. To more easily visualize the data, the protein data were expressed as a ratio of water deficit to control values of protein abundance in individual heat maps in mulitple metabolic pathways, including photosynthesis-photorespiration (Figure 7), glycolysis (Figure 8), the TCA cycle and amino acid metabolism (Figure 9), ascorbate-glutathione metabolism (Figure 10) and phenylalanine biosynthesis (Figure 11). When multiple isozymes were present, the heat maps represent summaries of multiple isozymes when their patterns were similar. In other cases, when there were obvious differences in expression between isozymes, two of the most contrasting heat map patterns were displayed. The contrasting isozymes likely represent enzymes in different locations and/or functions. For example, sucrose synthase (2.4.1.13; see Figure 8) in glycolysis is involved in sucrose catabolism in the cytoplasm, and also in cellulose biosynthesis at the inner surface of the plasma membrane. If no heat map is present for the enzyme then there were no data for this protein.

### Photosynthesis and photorespiration

The abundance of proteins involved in photosynthesis and photorespiration were strongly affected by water deficit (Figure 7). The abundance of many photosynthesis proteins were increased by water deficit on Day 4, whereas proteins involved in photorespiration were increased in abundance on Day 12. One of the most strongly affected proteins was the ferredoxin-NADP reductase (EC 1.18.1.2) located on the thylakoid membranes at the end of the Photosystem I (PS I) electron transport chain. The abundance of this protein fitted into Cluster 1 and was increased by water deficit by Day 4 and remained elevated compared to its control throughout the duration of the water deficit experiment. Othe proteins directly interacting or closely interacting with ferredoxin were also highly increased by water deficit including thioredoxin-f (Trx-f), superoxide dismutase (1.15.1.1), catalase (1.11.1.6), and peroxiredoxin (1.11.1.15). Glyceraldehyde-3-phosphate dehydrogenase (1.2.1.12) and sedoheptulose 1,7-bisphosphatase (3.1.3.37) of the Calvin-Benson cycle also had strong responses to water deficit. The abundance of most of the other photosynthetic proteins in the Calvin-Benson cycle were increased by water deficit on Day 4 but with varying patterns overtime (Figure 7). The abundance of the photorespiration proteins, glycolate oxidase (1.1.3.15), glycine dehydrogenase (1.4.4.2) and serine-glyoxylate aminotransferase (2.6.1.45) were increased on Day 12 by water deficit (Cluster 3). However, two isozymes of serine hydroxymethyltransferase (2.1.2.1) were increased on Day 4: D7T5C1 in Cluster 1 and F6GZK4 in Cluster 3; one isozyme, F6GWF3, was decreased on Day 12 (Cluster 4).

The increase of five key enzymes of the Calvin-Benson cycle indicates regulation by the ferredoxin-thioredoxin system [30]. Consistent with this, the abundance of a thioredoxin-f type protein (Trx-f, D7WPN1, additional file 1) was significantly increased throughout the water deficit treatment (Figure 7). Note that this protein was not included in the 942 protein dataset because peptides were absent from one of the replicates of the control, Day 16 treatment. Nevertheless the response was statistically significant. Trx-f can regulate the enzymes of the Calvin-Benson cycle [30]. Trx-f is also known to activate peroxiredoxins (1.11.1.15), which were highly increased throughout water deficit as well (Figure 7). Thus, some of the earliest and largest responses to water deficit appear to be localized around the activities of ferredoxin at the end of the electron transport chain of PS I. The activities are increased prior to any detectable decreases in physiological responses (growth and photosynthesis).

### Glycolysis

Glycolysis is important for the production of energy and carbon skeletons of primary and secondary metabolites. Water deficit had significant effects on proteins involved in glycolysis (Figure 8). The abundance of a number of enyzmes were increased (hexokinase-1, glyceraldehye-3-phosphate dehydrogenase, triose phosphate isomerase, phosphoglycerate kinase, malate dehydrogenase and malic enzyme) or decreased (phosphoglycerate mutase, phosphoglucomutase, phosphofructokinase, and pyruvate kinase) on Day 4. There are alternate forms of glycolysis [20,21,31,32] used in different physiological conditions. Pyruvate kinase plays a critical role in the regulation of glycolysis [32]. The large decrease in pyruvate kinase is consistent with a stimulation of an alternate pathway of glycolysis through phosphoenolpyruvate carboxylase (PEPC), malate dehydrogenase and malic enzyme (Figure 8). This alternate pathway of glycolysis is thought to be more efficient when plants are ADP-limited during stress [20,33]. In addition, PEPC is very important for the replenishment of metabolites to the tricarboxylic acid cycle (TCA) cycle that are used for biosynthetic processes [31,33].

### The TCA cycle and the biosynthesis of glutamate, threonine, glycine and cysteine

The TCA cycle is a central hub in plant metabolism, giving rise to many primary and secondary metabolites including several intermediates involved in amino acid biosynthesis and nitrogen assimilation [34]. The protein abundance of most of the enzymes of the TCA cycle were increased by water deficit (Figure 9). Two keys steps for amino acid biosynthesis are the production of 2-oxoglutarate by isocitrate dehydrogenase and oxaloacetate by malate dehydrogenase giving rise to glutamate and aspartate, respectively. These enzymes are significantly increased by water deficit (Figure 9). Glutamate is an important intermediate for other amino acids, purines, glutathione, chlorophyll and catalase (Figure 9). Glutamate synthase (1.4.7.1; GOGAT) is a key step in nitrogen assimilation [34] and its abundance was very elevated by water deficit. Interestingly, GOGAT uses reduced ferredoxin from PS I (also having an elevated protein abundance as described above) to convert one oxoglutarate and glutamine to two glutamates. The important antioxidant, glutathione, is a tripeptide synthesized from glutamate, cysteine and glycine. The abundance of proteins involved in the biosynthesis of all three of these amino acids was increased by water deficit. The protein abundance of threonine synthase (4.2.3.1) was highly increased by water deficit on Day 4 only. Threonine is used in the synthesis of glycine and pyridoxine (Vitamin B6); pyridoxine can act as an antioxidant and can protect abiotically-stressed plants [35,36]. Note that the protein abundance of homoserine kinase (2.7.1.39) in the threonine biosynthesis pathway was highly increased by water deficit throughout the stress period. The serine biosynthesis pathway from 3-phosphoglycerate (see 3-phosphoglycerate dehydrogenase; 1.1.1.95) was downregulated by Day 12 of the water deficit, but this reduction might be compensated by the increased protein abundance of serine hydroxymethyltransferase (2.1.2.1). Consistent with these proteomics results, water deficit increases the abundance of serine, glycine and glutamate in the shoot tips of grapevine [9]. Interestingly, proteins involved in purine metabolism were decreased in abundance (Additional file 1), perhaps contributing to a decrease in consumption of glutamate for this pathway and increasing glutamate supply for other pathways.

### Ascorbate-glutathione metabolism

Consistent with the increased abundance of proteins involved with the biosynthesis of glycine, glutamate and cysteine and the H_2_O_2_ scavengers, catalase and peroxiredoxins, the abundance of antioxidant defense proteins in the ascorbate-glutathione cycle were increased (Figure 10). Ascorbate peroxidase (1.11.1.11) was highly elevated at Day 4 and remained elevated compared to control there after. Glutathione peroxidase (1.11.1.9) was increased at Day 4 and fits into Cluster 3 with increasing elevation of abundance over time (except on Day 8). Both of these enzymes scavenge H_2_O_2_. Longer term effects at Day 12 appear to contribute to longer term production of ascorbate by increasing galactose metabolism to produce ascorbate and decreasing tartrate biosynthesis to decrease ascorbate catabolism (Figure 10). The protein abundance of a UDP-glucose epimerase (5.1.3.2), which may be involved in the biosynthesis of galactose was increased and idonate dehydrogenase (1.1.1.264), a key step in ascorbate conversion to tartrate [37], was decreased by water deficit.

### Translation

Proteins involved in translation were decreased in abundance by water deficit (Cluster 2 and 4). There was a decrease of the protein abundance of six ribosomal proteins (A5ASC2, A5BUA6, A5BIA1, A5BHY1, F6HTT2, and F6GXM7) and one argynyl-tRNA synthetase (F6HGX8) on Day 4. These proteins are involved in translation and precede the detectable inhibition of shoot elongation by water deficit on Day 6 (see Cluster 2 proteins in Additional file 5 and Figure 4). The abundance of additional proteins involved in translation were identified in Cluster 4 (Additional file 7). These include 19 ribosomal proteins, 6 tRNA synthetases, 5 elongation factors, 3 subunits of eukaryotic translation initiation factor 3, a nascent polypeptide-associated complex (NAC), an alpha subunit family protein, and the GCN1 translational activator.

### Phenylpropanoid biosynthesis

Phenylalanine is an important precursor of phenylpropanoids. The abundance of proteins involved in the shikimate pathway and the biosynthesis of phenylalanine were significantly decreased by water deficit (Figure 11). Phospho-2-dehydro-3-deoxyheptonate aldolase (2.5.1.54) is the first critical step for the shikimate pathway and phenylpropanoid biosynthesis [38] and its abundance was decreased significantly on Day 4 and throughout the water deficit (Figure 11). Phenylpropanoids are used in lignin biosynthesis, which represents a significant pathway (sink) for C and energy flow [38]. This pathway may be decreased to conserve C skeletons and energy for other pathways. Consistent with this hypothesis, lignin content was found to be decreased in drought-stressed maize leaves [39]. In addition, the abundance of chalcone synthase was decreased more than any other protein by water deficit (Additional file 1). Chalcone synthase is one of the key proteins regulating the biosynthesis of flavonoids, a subclass of phenylpropanoids that are involved in defense responses [40].

### Summary of proteomic responses

**Figure 12:**
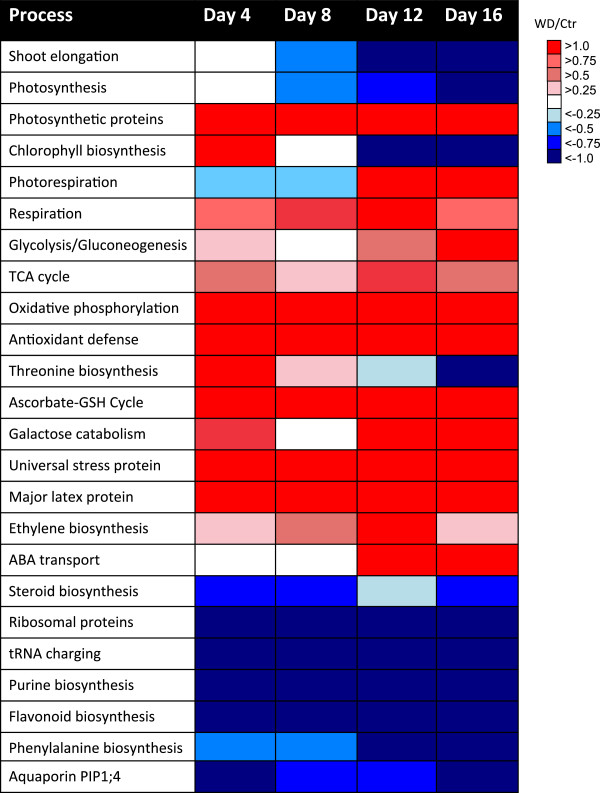
**A summary of the effects of water deficit on physiological and metabolic processes over time.** The boxes represent the ratio of water deficit treatment (WD) to control treatment (Ctr) values of each process as defined in the key. Boxes from left to right are ratios for Days 4 through 12.

A summary of the proteomic responses of expanding grapevine shoot tips to water deficit is presented in Figure 12 to permit easy comparisons of the changes in the processes with time. These changes are displayed as a heat map for easy visualization. Physiological responses start at the top of the figure followed by increased and decreased biochemical processes, respectively. One of the earliest responses to water deficit was the up-regulation of photosynthesis and antioxidant defenses. In addition there were simultaneous decreases in growth-related processes, such as protein synthesis (translation), brassinosteroid biosynthesis and water transport. These growth related processes very likely led to decreased cell wall biosynthesis, redirecting C flow to other pathways. Consistent with this hypothesis was the decreased abundance of two different cellulose synthase A subunits (F6H311 and F6HB61; Additional file 1) by water deficit, which completely disappeared on Day 12 and Day 16 of the stress treatment (the abundance was below the level of detection). Recall that one of the isozymes of sucrose synthase, which may be a part of the cellulose biosynthetic apparatus, was also decreased by water deficit (see Figure 8). Two stress-responsive proteins, a universal stress protein (F6H727) and a major latex protein (F6HFH0) were also noteworthy; their abundance was highly increased by water deficit from Day 4 to Day 16. Two stress hormone responses in addition to the brassinosteroids response were also detected. The abundance of an ACC oxidase, which is the last step in ethylene biosynthesis was increased throughout the water deficit and the abundance of an abscisic acid (ABA) transporter was increased on Day 12 and 16 of the water deficit treatment.

## Discussion

Proteomic analyses in this study indicated that the proteome was dynamic, changing early in response to water deficit. More than 200 protein profiles changed prior to any detectable changes in growth or photosynthesis in response to water deficit.

### Inhibition of growth

The inhibition of shoot or cell elongation is one of the most sensitive responses to water deficit and generally precedes the inhibition of photosynthesis [41-47]; however, in this study we could not distinguish the difference between the initial start in the decrease of growth and photosynthesis; growth and photosynthesis appeared equally sensitive to water deficit. Unlike osmotic shock treatments, the gradual application of stress in this experiment enabled the gradual progression of events to be evaluated. In three previous reports [8,9,47], shoot elongation was shown to be very sensitive to water deficit, decreasing before decreases in stem water potential were statistically detectable. Water-deficit at equivalent water potentials has a greater initial impact than salinity on shoot elongation of grapevines [9].

Interestingly, there was an early decrease in abundance of the brassinosteroid biosynthesis protein, Diminuto/DWF1(Day 4; Figure 5), preceding the inhibition of shoot elongation by water deficit (Day 6; Figure 1). This is a novel and intriguing observation. Knockouts of the Diminuto/DWF1 gene cause a dwarf phenotype in *Arabidopsis*[18]. The Diminuto/DWF1 gene was cloned in grapes and shown to be associated with brassinosteroid biosynthesis in grape berries [48]. It is not known if steroid concentrations change in response to water deficit in grapevine. Another protein that may affect growth was the decrease in protein abundance of an aquaporin (PIP1;4) on Day 4 that may regulate water uptake into the shoot tip.

### Inhibition of translation

Like growth, protein synthesis is very sensitive to water deficit [41,49-52], but it has been difficult to assess whether or not a decrease in growth preceded a decrease in translation or vice versa. In the present study there was a clear decrease of the protein abundance of multiple ribosomal proteins and an aminoacyl t-RNA synthase that are involved in translation on Day 4. Again, the decrease in protein abundance preceded the detectable inhibition of shoot elongation by water deficit on Day 6 (see Cluster 2 proteins in Additional file 5 and Figure 4).

### Effects on photosynthesis

In the present study, the abundance of numerous photosynthesis-related proteins was increased (Additional file 4 and Figure 7) by Day 4 prior to the inhibition of photosynthesis (Figure 1). Note some caution in interpretation of these data is warranted, because proteins and photosynthesis were measured on different leaves. The proteins were extracted from the growing shoot tip which contained immature leaves that were largely acting as sinks, however some of these leaves of the growing shoot tip were most likely active in photosynthetic function and probably responded in a similar manner as the fully mature leaves. The photosynthesis measurements were measured on the first, fully-expanded leaf adjacent to the growing tip, acting as a source rather than a sink. Many of the identified photosynthetic proteins in the growing shoot tip function in light harvesting, carbon fixation, photosystem II, photosystem repair, and the regulation of light reactions.

The transcript profiles of most of the genes encoding these proteins in the shoot tip did not correlate with the protein profiles (unpublished results). In fact, there were no statistically significant changes induced by stress in any transcript profiles until Day 8 in a previous experiment [9], when growth is significantly inhibited and stem water potentials have significantly declined. The data indicate that the early changes in protein abundance of photosynthetic enzymes are involved in photosynthetic maintenance and repair and that no real impairment of photosynthesis is occurring at that time (an elastic strain rather than a plastic strain as defined by Levitt [53]). This argument is supported by a short-term stress experiment comparing chilling to osmotic stress in the shoot tips of Cabernet Sauvignon [54], which indicated that photosynthetic mechanisms could be repaired in a short-term osmotic stress, but repair mechanisms were inhibited in a short-term chilling stress.

Similar metabolic acclimation occurs in Arabidopsis [45]. While there were no significant decreases in photosynthesis by mild water stress, because of the decreased growth there was an actual increase in carbon availability for the production of low molecular weight metabolites such as proline, organic acids (malate and fumarate) and hexoses. Furthermore, enzyme activities were elevated by water deficit to maintain photosynthesis and glycolysis [45].

Water stress inhibits photosynthesis in two stages [55]. In the first stage, photosynthesis is primarily limited by CO_2_ diffusion. In the second stage, as the stress becomes more severe, metabolic inhibition occurs. Associated with this metabolic impairment is an increase in the activity of antioxidant mechanisms [56]. In the present proteomics study, clear increases in antioxidant defenses were detected on Day 4, well before a decline in photosynthesis or growth. In a previous transcriptomics and metabolomics study [9], a clear increase in antioxidant mechanisms occurred starting at Day 12. In the transcript and metabolite profiles of the previous study, water-deficit-stressed plants were more strongly affected than salt-stressed plants. The data indicate that plants exposed to water-deficit are subject to more reactive oxygen species (ROS) and photoinhibition than salt-stressed plants. It appears that the increase in the metabolites, glucose, glutamate, glycine, proline and malate, and the large increase in transcripts involved in photorespiration and antioxidant proteins at Day 12 facilitate osmotic adjustment, ROS detoxification and protection against photoinhibition in these severely water-stressed plants. In particular, glutamate appears to be a central metabolite in this response as it is used in many metabolic pathways involved in the grapevine response to water deficit including amino acid metabolism (γ-amino-butyric acid (GABA), proline, glycine, etc.), nitrogen assimilation, and the biosynthesis of catalase, glutathione, and chlorophyll. Glutamate metabolic flux appears to drive GABA production more than proline in water-deficit stressed tobacco and GABA provides greater protection against ROS than proline [57].

### Adjustments in glycolysis

Environmental stresses can cause subtle adjustments in glycolysis in plants [20,45]. Two major functions of glycolysis are to produce energy and the building-blocks needed for biochemical synthesis. There are multiple ways that this pathway can be finely controlled including pH, substrate concentrations, metabolite effector concentrations and protein modification [20]. Such adjustments in glycolysis have been observed with hypoxia and nutrient deprivation [20,32,33]. We could not find any studies on the effect of water deficit on an alternate pathway of glycolysis involving pyruvate kinase and PEPC. In arabidopsis leaves, steady-state water deficit increased the activity of pyruvate kinase and PEPC [45]. Overall activities in the enzymes of the TCA cycle were maintained during water deficit, but alternate paths of glycolysis were not discussed. There was an increase in C supply in the plants, which was attributed to a reduction of growth before photosynthesis. Alternative oxidase in respiration is another alternate pathway affected by water deficit [58,59]. The changes in an alternate glycolysis observed here in grapevine may allow energy to be used more efficiently [20] and may contribute to an increased flux of glutamate through various metabolic pathways.

### Protein activity

Protein amount does not necessarily equate to protein activity. Many proteins are affected by post-translational modifications such as phosphorylation. A large number of key regulatory proteins in central C and amino acid metabolism have altered phosphorylation states depending on environmental conditions [60]. Furthermore, there are significant changes in the phosphoproteome of growing maize leaves in response to water deficit [47]. Many of the proteins detected in the present grapevine study are regulated by phosphorylation including hexokinase, phosphofructokinase, pyruvate kinase, PEPC, citrate synthase, threonine synthase, and 3-phosphoglycerate dehydrogenase to name but a few. Therefore additional investigations are necessary to determine the phosphorylation status of these enzymes and their activity to further clarify their role in metabolism during water deficit.

### Comparisons to other plant proteomics studies of water deficit

There are quite a few studies on the proteomics response of plants to water deficit [61] most of which have been performed with 2-D gel electrophoresis. Several studies indicate an effect of water deficit on photosynthetic enzymes and C metabolism [8,62-65], antioxidant defenses [66-71], and protein synthesis [72].

In grapevine, a proteomics analysis of water deficit and salinity on shoot tips [8] showed that the inhibition of shoot elongation was correlated with the inhibition of proteins involved in photosynthesis, protein synthesis and protein fate. Many of the enzymes affected by water deficit in the present study were not affected significantly by water deficit in the previous study. The lack of significance may be due to the relatively large CV found when using 2-D PAGE, which has an average CV of approximately 50% (unpublished results), wherease the CV in the present study using nanoLC-MS/MS was only 9%. The high CV makes it much more difficult to detect statistical significance even though the treatment effects may be real.

In drought-stressed grape berry skins [73], similar to the present study on shoot tips, long-term water deficit increased the abundance of ascorbate peroxidase. In the berry pulp, the abundance of glutamate decarboxylase was increased by water deficit. Glutamate decarboxylase was decreased by water deficit (Cluster 4) in shoot tips in the present study. The study on berry tissues indicates that there are differential responses to water deficit depending on the tissue; this is likely related to the function of the tissue or organ.

One proteomics study shows clear root signaling in rice in response to water deficit [71]. A split-root design was used to analyze the effects of water deficit on both the well-watered and water-deficit treated roots. A number of proteins were up-regulated in the well-watered split roots of rice as compared to ordinary well-watered roots; that is the well-watered split roots were receiving signals from the water-deficit-treated split roots. One of the proteins up-regulated in the well-watered split roots was a universal stress protein and a number of proteins involved in ROS defense, including several in the ascorbate-glutathione cycle. This is very similar to the early responsive proteins of grapevine shoot tips in the present study.

There are significant changes in the xylem sap proteome of maize in response to water deficit [74]. Proteins associated with cell wall metabolism and biotic defense response increased in abundance in response to water deficit after changes in stomatal conductance including cell wall peroxidases. Interestingly, several metabolites changed early in the xylem sap before decreasing stomatal conductance including ABA, malate and some amino acids.

In a very similar experimental design to the present study, the abundance of phosphoproteins in growing maize leaves was affected by mild and severe water deficit [47]. One difference in the experimental design to the present study was that the first sampling occurred soon after growth inhibition, not before. In addition, the rate of dehydration was faster than the present study with mild water deficit occurring after 1 day and severe water deficit occurring after 4 days. In addition, a rehydration response was also investigated in the maize study. The phosphoproteomics response to water deficit and rehydration was very dynamic, affecting 132 out of 1235 reproducible phosphopeptides. The top three functional categories affected were chromosome remodeling, cell expansion and phytohormone signaling, respectively. Early stress responsive phosphoproteins were involved in cell division and cell wall biosynthesis. Upon rewatering, there were rapid responses in phosphoproteins involving ABA, ethylene and jasmonate signaling.

### Speculation on root signals and feedforward mechanisms

The change in protein abundance prior to any detectable changes in physiology in response to water deficit in the present grapevine study may indicate a feedforward mechanism caused by hormone and/or hydraulic signals from the root [75-80]. Tall and narrow tree pots were used in this study. These pots would allow a water gradient to form in the pot; the upper roots would dry out first. Roots in the bottom of the pot, where most of the roots are located, would continue transporting water to the shoot tips to supply enough water for shoot elongation. The signal would have been produced in the root tips that were drying out in the upper portion of the pot.

ABA has been described as such a feedforward signal from drying root tips that prematurely closes stomates to reduce water loss from the soil in advance of water deficit [79,80], however in the present study stomatal conductance decreased after changes in protein metabolism. A hydraulic signal due to a changing water potential gradient [77,80,81] is another possibility, however, this gradient would have to be small, because it did not significantly affect shoot elongation. A third possibility is a long-distance ROS signal that was elegantly demonstrated to respond rapidly to various biotic and abiotic stresses [82,83]. It remains to be demonstrated if slow drying of root tips could trigger such a signal. It is possible that ferredoxin may be an early target of such ROS signaling because the abundance of a number of antioxidant proteins that interact with ferredoxin were some of the most highly increased by water deficit on Day 4. Future experiments with greater time resolution are needed to test these signaling hypotheses.

## Conclusions

In summary, the analyses of protein profiles have provided a deeper insight into the response of water-deficit-stressed plants. In this study, the quantitative proteomic analysis of grapevine shoot tips exposed to water deficit indicated that stress affected many proteins involved in metabolism, energy (photosynthesis, glycolysis and respiration), antioxidant defense and protein fate (protein synthesis, folding and degradation). Many of these changes may represent acclimation responses, occurring prior to any detectable changes in shoot elongation and photosynthesis. Early changes in protein abundance in response to stress occurred with photosynthesis, translation-, growth- and C metabolism that might be affected by phosphorylation. Many other proteins are known to be regulated by ROS and ferredoxin-thioredoxin signaling. These early and massive proteomic responses to water deficit indicate that there was an increase in C metabolism and energy production, shifting away from growth and toward protective antioxidant defenses. Longer-term effects on protein abundance indicated that more severe water deficit impacted additional processes involving photorespiration, stress responses, protein fate and cellular defense.

## Methods

### Plant material and experimental conditions

Plant material and experimental conditions along with physiological assays have been described in detail in previous publications [5,9]. Briefly, two-year-old rooted cuttings of dormant *Vitis vinifera* cv. Cabernet Sauvignon clone 8 were grown in 13.3 liter pots containing approximately 10 L SuperSoil® potting mix supplemented with slow release fertilizer. Plants were grown in a greenhouse on a 16 h light (26°C, minimum 400 μE m^-2^ s^-1^)/8 h dark (18°C) cycle supplemented with illumination from sodium vapor lamps. Vines were irrigated daily with tap water (control) or not irrigated at all (water deficit).

Growth was measured repeatedly on five independent biological replicates that were harvested 16 days after the start of the experiment. Elongating shoot tips of 3 individual biological replicates for each time point (Day 4, 8, 12, and 16) and treatment (control and water deficit) were harvested and frozen immediately in liquid nitrogen in preparation for protein extraction. Elongating shoot tips consisted of the shoot apex, stems, tendrils and the first four immature leaves. Photosynthesis and stomatal conductance were measured with a LI-6400XT portable photosynthesis system (LI-COR, Lincoln, NE, USA) on the first fully expanded leaf (leaf number 6 from the apex) adjacent to the shoot tip.

Proteins were extracted from frozen, finely-ground shoot tip samples using a phenol-based extraction protocol [10]. In preparation for gel-free shotgun proteomics, protein pellets were Lys-C- and trypsin-digested using a modified method (A. Apffel, personal communication) of the Filter-Aided Sample Preparation (FASP) methods [84,85]. The modified FASP uses trifluoroethanol (TFE) instead of urea to denature proteins. Protein extracts (250 μg each) were dissolved in 200 μL 50% TFE, 0.1 M ammonium bicarbonate, 50 mM dithiothreitol (DTT) and concentrated to ~20 μL in Amicon Ultra 0.5 mL 10K ultrafiltration devices (Millipore). Then 100 μL 50% TFE, 0.1 M ammonium bicarbonate (ABC), 50 mM iodoacetamide was added to the devices, incubated at room temperature 1 h, followed by centrifugation (14,000 *g*, 45 min). The alkylated proteins were then washed five times in the ultracentrifugation devices with 200 μL 50% TFE, 0.1 M ABC. To the ~20 μL retentates, 45 μL 50% TFE, 0.1 M ABC was added, then 2.5 μg Lys-C (Wako) from a 0.5 μg/μL stock solution. Following overnight incubation at 28°C, 350 μL 20% acetonitrile (CAN), 50 mM ABC was added to each digest, followed by 2.5 μL of 1 μg/μL modified trypsin (Promega). The trypsin digests took place at 37°C for two hours, then they were stopped with 10 μL 50% formic acid. The resulting peptides were passed through the ultrafiltration membranes by centrifugation (14,000 *g*, 45 min), followed by a 150 μL rinse of the UF devices with 50% ACN, 2% formic acid. The peptide filtrates were concentrated by Speedvac to near-dryness, then reconstituted in 12.5 μL 40% formic acid, 10% TFE and diluted for LC-MS/MS with 60 μL water.

LC-MS/MS spectra were acquired from 3 biological replicates per treatment and timepoint by a sample-optimized gas phase fractionation (GPF) method (similar to [86]) on an LTQ XL mass spectrometer (Thermo). Chromatography was performed on a Surveyor autosampler/LC system (Thermo) and 0.3 x 150 mm PLRP-S column (3 μm, 100 Å, Agilent) interfaced with the mass spectrometer by an Advance captive spray source (Michrom). Each sample (10 μL injections) was separated by four 180 min LC-MS/MS runs at 5 μL min^-1^ differing only in precursor m/z ranges. The m/z ranges for four gas phase fractions per sample were optimized empirically by analyzing a mixture of pooled samples from m/z 400–2000, then creating GPF fractions to approximately evenly distribute peptide observations among the four fractions. Using data dependent acquisition of MS/MS spectra of the most abundant six peaks per MS scan (with dynamic exclusion), the analysis time of 12 h per sample produced approximately 118,000 MS/MS spectra per sample.

A protein database was compiled from three sources 10 September 2011: 1) all reviewed *V. vinifera* protein entries in UniProt (151 sequences); 2) *V. vinifera* proteins predicted by the International Grape Genome Program (29749 sequences identified by UniProt search “Taxonomy:29760 AND author:vitulo AND reviewed:no”); 3) mitochondrial proteins associated in UniProt with [87] and [88] (81 non-redundant sequences).

Spectrum-peptide matching was performed with X!Tandem and the GPM Cyclone (http://www.thegpm.org) in automated mode using MudPit merging. Default ion trap parameters were used with the exceptions of MS error (+3, -1 Da), the inclusion of point mutations, the inclusion of reversed sequences, and a protein expect value of −1. Approximately 27,000 spectra per sample were assigned to peptides.

Normalized spectral abundance factors (NSAF) were calculated according to Zybailov et al. [89]. Protein identifications were filtered and protein and peptide FDRs calculated according to Gammulla et al. [90]. Protein identifications were excluded if they did not match at least one spectrum in all three biological replicates and at least a total of six spectra among the replicates. The protein false discovery rate was 0.57% and the peptide false discovery rate was 0.056%. The mass spectrometry proteomics data have been deposited to the ProteomeXchange Consortium (http://proteomecentral.proteomexchange.org) via the PRIDE partner repository [91] with the dataset identifier PXD000123.

## Competing interests

The authors declare that they have no competing interests.

## Authors’ contributions

GRC conceived and conducted the experiment, analyzed all of the data and wrote the body of the paper; SVS digested the proteins and collected and analyzed the proteomics data; DWH assisted in running the growth experiment and the analysis of the data; DP and TK assisted in the statistical analyses; PAH supported and assisted in the data analysis. All authors reviewed, edited and approved the final version of the manuscript.

## Supplementary Material

Additional file 1 Proteomic details of all proteins identified in shoot tips of Cabernet Sauvignon. Click here for file

Additional file 2 BinGO results for overrepresented GO biological process functional categories for all proteins identified. Click here for file

Additional file 3 Statistical results for all significantly affected proteins in the all present (942 proteins) dataset based on treatment and treatment x day interaction. Click here for file

Additional file 4 BinGO results for overrepresented GO biological process functional categories in Cluster 1. Click here for file

Additional file 5 BinGO results for overrepresented GO biological process functional categories in Cluster 2. Click here for file

Additional file 6 BinGO results for overrepresented GO biological process functional categories in Cluster 3. Click here for file

Additional file 7 BinGO results for overrepresented GO biological process functional categories in Cluster 4. Click here for file

## References

[B1] Cramer GR Urano K Delrot S Pezzotti M Shinozaki K Effects of abiotic stress on plants: a systems biology perspective BMC Plant Biol 2011 11 163 10.1186/1471-2229-11-163 22094046 PMC3252258

[B2] Munns R Comparative physiology of salt and water stress Plant Cell Environ 2002 25 2 239 250 10.1046/j.0016-8025.2001.00808.x 11841667

[B3] Greenway H Munns R Mechanisms of salt tolerance in nonhalophytes Annu Rev Plant Physiol 1980 31 149 190 10.1146/annurev.pp.31.060180.001053

[B4] Tester M Davenport R Na^+^ tolerance and Na^+^ transport in higher plants Ann Bot 2003 91 5 503 527 10.1093/aob/mcg058 12646496 PMC4242248

[B5] Tattersall EA Grimplet J Deluc L Wheatley MD Vincent D Osborne C Ergul A Lomen E Blank RR Schlauch KA Cushman JC Cramer GR Transcript abundance profiles reveal larger and more complex responses of grapevine to chilling compared to osmotic and salinity stress Funct Integr Genomics 2007 7 4 317 333 10.1007/s10142-007-0051-x 17578611

[B6] Kreps JA Wu Y Chang HS Zhu T Wang X Harper JF Transcriptome changes for Arabidopsis in response to salt, osmotic, and cold stress Plant Physiol 2002 130 4 2129 2141 10.1104/pp.008532 12481097 PMC166725

[B7] Seki M Narusaka M Ishida J Nanjo T Fujita M Oono Y Kamiya A Nakajima M Enju A Sakurai T Satou M Akiyama K Taji T Yamaguchi-Shinozaki K Carninci P Kawai J Hayashizaki Y Shinozaki K Monitoring the expression profiles of 7000 Arabidopsis genes under drought, cold and high-salinity stresses using a full-length cDNA microarray Plant J 2002 31 3 279 292 10.1046/j.1365-313X.2002.01359.x 12164808

[B8] Vincent D Ergul A Bohlman MC Tattersall EA Tillett RL Wheatley MD Woolsey R Quilici DR Joets J Schlauch K Schooley DA Cushman JC Cramer GR Proteomic analysis reveals differences between *Vitis vinifera* L. cv. Chardonnay and cv. Cabernet Sauvignon and their responses to water deficit and salinity J Exp Bot 2007 58 7 1873 1892 10.1093/jxb/erm012 17443017

[B9] Cramer GR Ergul A Grimplet J Tillett RL Tattersall EA Bohlman MC Vincent D Sonderegger J Evans J Osborne C Quilici D Schlauch KA Schooley DA Cushman JC Water and salinity stress in grapevines: early and late changes in transcript and metabolite profiles Funct Integr Genomics 2007 7 2 111 134 10.1007/s10142-006-0039-y 17136344

[B10] Vincent D Wheatley MD Cramer GR Optimization of protein extraction and solubilization for mature grape berry clusters Electrophoresis 2006 27 9 1853 1865 10.1002/elps.200500698 16586412

[B11] Saeed AI Bhagabati NK Braisted JC Liang W Sharov V Howe EA Li J Thiagarajan M White JA Quackenbush J TM4 microarray software suite Methods Enzymol 2006 411 134 193 16939790 10.1016/S0076-6879(06)11009-5

[B12] Lundin B Hansson M Schoefs B Vener AV Spetea C The Arabidopsis PsbO2 protein regulates dephosphorylation and turnover of the photosystem II reaction centre D1 protein Plant J 2007 49 3 528 539 10.1111/j.1365-313X.2006.02976.x 17217465

[B13] Taiz L Zeiger E Plant Physiology, 5th ed 2010 SinauerAssociates, Inc: Sunderland, MA

[B14] Vandeleur RK Mayo G Shelden MC Gilliham M Kaiser BN Tyerman SD The role of PIP aquaporins in water transport through roots: diurnal and drought stress responses reveal different strategies between isohydric and anisohydric cultivars of grapevine Plant Physiol 2009 149 1 445 460 10.1104/pp.108.128645 18987216 PMC2613730

[B15] Gambetta GA Manuck CM Drucker ST Shaghasi T Fort K Matthews MA Walker MA McElrone AJ The relationship between root hydraulics and scion vigour across Vitis rootstocks: what role do root aquaporins play? J Exp Bot 2012 63 18 6445 6455 10.1093/jxb/ers312 23136166 PMC3504504

[B16] Klahre U Noguchi T Fujioka S Takatsuto S Yokota T Nomura T Yoshida S Chua NH The Arabidopsis DIMINUTO/DWARF1 gene encodes a protein involved in steroid synthesis Plant Cell 1998 10 10 1677 1690 9761794 10.1105/tpc.10.10.1677 PMC143945

[B17] Jager CE Symons GM Ross JJ Reid JB Do brassinosteroids mediate the water stress response? Physiol Plant 2008 133 2 417 425 10.1111/j.1399-3054.2008.01057.x 18282191

[B18] Oliver MJ Jain R Balbuena TS Agrawal G Gasulla F Thelen JJ Proteome analysis of leaves of the desiccation-tolerant grass, Sporobolus stapfianus, in response to dehydration Phytochem 2011 72 10 1273 1284 10.1016/j.phytochem.2010.10.020 21109273

[B19] Carroll AJ Heazlewood JL Ito J Millar AH Analysis of the Arabidopsis cytosolic ribosome proteome provides detailed insights into its components and their post-translational modification Mol Cell Proteomics 2008 7 2 347 369 17934214 10.1074/mcp.M700052-MCP200

[B20] Plaxton WC The organization and regulation of plant glycolysis Annu Rev Plant Physiol Plant Mol Biol 1996 47 185 214 10.1146/annurev.arplant.47.1.185 15012287

[B21] Sweetman C Deluc LG Cramer GR Ford CM Soole KL Regulation of malate metabolism in grape berry and other developing fruits Phytochem 2009 70 11–12 1329 1344 10.1016/j.phytochem.2009.08.006 19762054

[B22] Mhamdi A Queval G Chaouch S Vanderauwera S Van BF Noctor G Catalase function in plants: a focus on Arabidopsis mutants as stress-mimic models J Exp Bot 2010 61 15 4197 4220 10.1093/jxb/erq282 20876333

[B23] Kang J Hwang JU Lee M Kim YY Assmann SM Martinoia E Lee Y PDR-type ABC transporter mediates cellular uptake of the phytohormone abscisic acid Proc Natl Acad Sci USA 2010 107 5 2355 2360 10.1073/pnas.0909222107 20133880 PMC2836657

[B24] Marton MJ Crouch D Hinnebusch AG GCN1, a translational activator of GCN4 in Saccharomyces cerevisiae, is required for phosphorylation of eukaryotic translation initiation factor 2 by protein kinase GCN2 Mol Cell Biol 1993 13 6 3541 3556 8497269 10.1128/mcb.13.6.3541-3556.1993 PMC359824

[B25] Viotti C Luoni L Morandini P De MMI Characterization of the interaction between the plasma membrane H-ATPase of Arabidopsis thaliana and a novel interactor (PPI1) FEBS J 2005 272 22 5864 5871 10.1111/j.1742-4658.2005.04985.x 16279950

[B26] Ho CL Noji M Saito M Saito K Regulation of serine biosynthesis in Arabidopsis. Crucial role of plastidic 3-phosphoglycerate dehydrogenase in non-photosynthetic tissues J Biol Chem 1999 274 1 397 402 10.1074/jbc.274.1.397 9867856

[B27] Zhang P Foerster H Tissier CP Mueller L Paley S Karp PD Rhee SY MetaCyc and AraCyc. Metabolic pathway databases for plant research Plant Physiol 2005 138 1 27 37 10.1104/pp.105.060376 15888675 PMC1104157

[B28] Ogata H Goto S Sato K Fujibuchi W Bono H Kanehisa M KEGG: Kyoto Encyclopedia of Genes and Genomes Nucleic Acids Res 1999 27 1 29 34 10.1093/nar/27.1.29 9847135 PMC148090

[B29] Grimplet J Cramer GR Dickerson JA Mathiason K Van Hemert J Fennell AY VitisNet: “Omics” integration through grapevine molecular networks PLoS One 2009 4 12 e8365 10.1371/journal.pone.0008365 20027228 PMC2791446

[B30] Buchanan BB Balmer Y Redox regulation: a broadening horizon Annu Rev Plant Biol 2005 56 187 220 10.1146/annurev.arplant.56.032604.144246 15862094

[B31] Tcherkez G Boex-Fontvieille E Mahe A Hodges M Respiratory carbon fluxes in leaves Curr Opin Plant Biol 2012 15 3 308 314 10.1016/j.pbi.2011.12.003 22244081

[B32] van Dongen JT Gupta KJ Ramirez-Aguilar SJ Araujo WL Nunes-Nesi A Fernie AR Regulation of respiration in plants: a role for alternative metabolic pathways J Plant Physiol 2011 168 12 1434 1443 10.1016/j.jplph.2010.11.004 21185623

[B33] O’Leary B Park J Plaxton WC The remarkable diversity of plant PEPC (phosphoenolpyruvate carboxylase): recent insights into the physiological functions and post-translational controls of non-photosynthetic PEPCs Biochem J 2011 436 1 15 34 10.1042/BJ20110078 21524275

[B34] Foyer CH Noctor G Hodges M Respiration and nitrogen assimilation: targeting mitochondria-associated metabolism as a means to enhance nitrogen use efficiency J Exp Bot 2011 62 4 1467 1482 10.1093/jxb/erq453 21282329

[B35] Havaux M Ksas B Szewczyk A Rumeau D Franck F Caffarri S Triantaphylides C Vitamin B6 deficient plants display increased sensitivity to high light and photo-oxidative stress BMC Plant Biol 2009 9 130 10.1186/1471-2229-9-130 19903353 PMC2777905

[B36] Chen H Xiong L Pyridoxine is required for post-embryonic root development and tolerance to osmotic and oxidative stresses Plant J 2005 44 3 396 408 10.1111/j.1365-313X.2005.02538.x 16236150

[B37] DeBolt S Cook DR Ford CM L-tartaric acid synthesis from vitamin C in higher plants Proc Natl Acad Sci USA 2006 103 5608 5613 10.1073/pnas.0510864103 16567629 PMC1459401

[B38] Novaes E Kirst M Chiang V Winter-Sederoff H Sederoff R Lignin and biomass: a negative correlation for wood formation and lignin content in trees Plant Physiol 2010 154 2 555 561 10.1104/pp.110.161281 20921184 PMC2949025

[B39] Vincent D Lapierre C Pollet B Cornic G Negroni L Zivy M Water deficits affect caffeate O-methyltransferase, lignification, and related enzymes in maize leaves. A proteomic investigation Plant Physiol 2005 137 3 949 960 10.1104/pp.104.050815 15728345 PMC1065396

[B40] Dao TT Linthorst HJ Verpoorte R Chalcone synthase and its functions in plant resistance Phytochem Rev 2011 10 3 397 412 10.1007/s11101-011-9211-7 21909286 PMC3148432

[B41] Bradford KJ Hsiao TC Lange OL, Nobel PS, Osmond CB, Ziegler H Physiological responses to moderate water stress Physiological Plant Ecology II 1982 12 B New York Berlin Heidelberg: Springer-Verlag 263 324

[B42] Cramer GR Alberico GJ Schmidt C Leaf expansion limits dry matter accumulation of salt-stressed maize Aust J Plant Physiol 1994 21 663 674 10.1071/PP9940663

[B43] Yeo AR Lee KS Izard P Boursier PJ Flowers TJ Short- and long-term effects of salinity on leaf growth in rice (*Oryza sativa* L.) J Exp Bot 1991 42 240 881 889

[B44] Saab IN Sharp RE Non-hydraulic signals from maize roots in drying soil: inhibition of leaf elongation but not stomatal conductance Planta 1989 179 4 466 474 10.1007/BF00397586 24201770

[B45] Hummel I Pantin F Sulpice R Piques M Rolland G Dauzat M Christophe A Pervent M Bouteille M Stitt M Gibon Y Muller B Arabidopsis plants acclimate to water deficit at low cost through changes of carbon usage: an integrated perspective using growth, metabolite, enzyme, and gene expression analysis Plant Physiol 2010 154 1 357 372 10.1104/pp.110.157008 20631317 PMC2938159

[B46] Tardieu F Granier C Muller B Water deficit and growth. Co-ordinating processes without an orchestrator Curr Opin Plant Biol 2011 14 3 283 289 10.1016/j.pbi.2011.02.002 21388861

[B47] Bonhomme L Valot BÆ Tardieu F Zivy M Phosphoproteome Dynamics Upon Changes in Plant Water Status Reveal Early Events Associated With Rapid Growth Adjustment in Maize Leaves Mol Cell Proteomics 2012 11 10 957 972 10.1074/mcp.M111.015867 22787273 PMC3494150

[B48] Symons GM Davies C Shavrukov Y Dry IB Reid JB Thomas MR Grapes on steroids. Brassinosteroids are involved in grape berry ripening Plant Physiol 2006 140 150 158 16361521 10.1104/pp.105.070706 PMC1326039

[B49] Ben-Zioni A Itai C Vaadia Y Water and salt stresses, kinetin and protein synthesis in tobacco leaves Plant Physiol 1967 42 361 365 10.1104/pp.42.3.361 16656515 PMC1086542

[B50] Flowers TJ Dalmond D Protein synthesis in halophytes: the influence of potassium, sodium and magnesium in vitro Plant Soil 1992 146 153 161 10.1007/BF00012008

[B51] Dhindsa RS Cleland RE Water stress and protein synthesis: I. Differential inhibition of protein synthesis Plant Physiol 1975 55 4 778 781 10.1104/pp.55.4.778 16659166 PMC541705

[B52] Hsiao TC Rapid Changes in Levels of Polyribosomes in Zea mays in Response to Water Stress Plant Physiol 1970 46 2 281 285 10.1104/pp.46.2.281 16657450 PMC396579

[B53] Levitt J Responses of plants to environmental stresses. Volume 1. Chilling freezing, and high temperature stresses 1980 New York: Academic Press

[B54] Ben Jouira H Schlauch KA Cushman JC Cramer GR Short-term abiotic stresses effects on proteomic profiles of Cabernet Sauvignon shoot tips BMC Plant Biol 2012 submitted

[B55] Flexas J Bota J Galmes J Medrano H Ribas-Carbo M Keeping a positive carbon balance under adverse conditions: responses of photosynthesis and respiration to water stress Physiol Plant 2006 127 3 343 352 10.1111/j.1399-3054.2006.00621.x

[B56] Cho K Shibato J Agrawal GK Jung YH Kubo A Jwa NS Tamogami S Satoh K Kikuchi S Higashi T Kimura S Saji H Tanaka Y Iwahashi H Masuo Y Rakwal R Integrated transcriptomics, proteomics, and metabolomics analyses to survey ozone responses in the leaves of rice seedling J Proteome Res 2008 7 7 2980 2998 10.1021/pr800128q 18517257

[B57] Liu C Zhao L Yu G The dominant glutamic acid metabolic flux to produce gamma-amino butyric acid over proline in Nicotiana tabacum leaves under water stress relates to its significant role in antioxidant activity J Integr Plant Biol 2011 53 8 608 618 10.1111/j.1744-7909.2011.01049.x 21564543

[B58] Rasmusson AG Fernie AR Van DJT Alternative oxidase: a defence against metabolic fluctuations Physiol Plant 2009 137 4 371 382 10.1111/j.1399-3054.2009.01252.x 19558416

[B59] Vanlerberghe GC Cvetkovska M Wang J Is the maintenance of homeostatic mitochondrial signaling during stress a physiological role for alternative oxidase? Physiol Plant 2009 137 4 392 406 10.1111/j.1399-3054.2009.01254.x 19549065

[B60] Oliveira AP Ludwig C Picotti P Kogadeeva M Aebersold R Sauer U Regulation of yeast central metabolism by enzyme phosphorylation Mol Syst Biol 2012 8 623 23149688 10.1038/msb.2012.55 PMC3531909

[B61] Kosova K Vitamvas P Prasil IT Renaut J Plant proteome changes under abiotic stress - Contribution of proteomics studies to understanding plant stress response J Proteomics 2011 74 8 1301 1322 10.1016/j.jprot.2011.02.006 21329772

[B62] Bonhomme L Monclus R Vincent D Carpin S Lomenech AM Plomion C Brignolas F Morabito D Leaf proteome analysis of eight Populus xeuramericana genotypes: genetic variation in drought response and in water-use efficiency involves photosynthesis-related proteins Proteomics 2009 9 17 4121 4142 10.1002/pmic.200900047 19722189

[B63] Kausar R Arshad M Shahzad A Komatsu S Proteomics analysis of sensitive and tolerant barley genotypes under drought stress Amino Acids 2012 44 2 345 359 22707152 10.1007/s00726-012-1338-3

[B64] Shu L Lou Q Ma C Ding W Zhou J Wu J Feng F Lu X Luo L Xu G Mei H Genetic, proteomic and metabolic analysis of the regulation of energy storage in rice seedlings in response to drought Proteomics 2011 11 21 4122 4138 10.1002/pmic.201000485 21818852

[B65] Bazargani MM Sarhadi E Bushehri AA Matros A Mock HP Naghavi MR Hajihoseini V Mardi M Hajirezaei MR Moradi F Ehdaie B Salekdeh GH A proteomics view on the role of drought-induced senescence and oxidative stress defense in enhanced stem reserves remobilization in wheat J Proteomics 2011 74 10 1959 1973 10.1016/j.jprot.2011.05.015 21621021

[B66] Hajheidari M Eivazi A Buchanan BB Wong JH Majidi I Salekdeh GH Proteomics uncovers a role for redox in drought tolerance in wheat J Proteome Res 2007 6 4 1451 1460 10.1021/pr060570j 17343403

[B67] Hajheidari M Abdollahian-Noghabi M Askari H Heidari M Sadeghian SY Ober ES Hosseini Salekdeh G Proteome analysis of sugar beet leaves under drought stress Proteomics 2005 5 4 950 960 10.1002/pmic.200401101 15712235

[B68] Sengupta D Kannan M Reddy AR A root proteomics-based insight reveals dynamic regulation of root proteins under progressive drought stress and recovery in Vigna radiata (L.) Wilczek Planta 2011 233 6 1111 1127 10.1007/s00425-011-1365-4 21298284

[B69] Wendelboe-Nelson C Morris PC Proteins linked to drought tolerance revealed by DIGE analysis of drought resistant and susceptible barley varieties Proteomics 2012 12 22 3374 3385 10.1002/pmic.201200154 23001927

[B70] Benesova M Hola D Fischer L Jedelsky PL Hnilicka F Wilhelmova N Rothova O Kocova M Prochazkova D Honnerova J Fridrichova L Hnilickova H The physiology and proteomics of drought tolerance in maize: early stomatal closure as a cause of lower tolerance to short-term dehydration? PLoS One 2012 7 6 e38017 10.1371/journal.pone.0038017 22719860 PMC3374823

[B71] Mirzaei M Soltani N Sarhadi E Pascovici D Keighley T Salekdeh GH Haynes PA Atwell BJ Shotgun Proteomic Analysis of Long-distance Drought Signaling in Rice Roots J Proteome Res 2012 11 1 348 358 10.1021/pr2008779 22047206

[B72] Mohammadi PP Moieni A Hiraga S Komatsu S Organ-specific proteomic analysis of drought-stressed soybean seedlings J Proteomics 2012 75 6 1906 1923 10.1016/j.jprot.2011.12.041 22245419

[B73] Grimplet J Wheatley MD Jouira HB Deluc LG Cramer GR Cushman JC Proteomic and selected metabolite analysis of grape berry tissues under well watered and water-deficit stress conditions Proteomics 2009 9 2503 2528 10.1002/pmic.200800158 19343710 PMC4090949

[B74] Alvarez S Marsh EL Schroeder SG Schachtman DP Metabolomic and proteomic changes in the xylem sap of maize under drought Plant Cell Environ 2008 31 3 325 340 10.1111/j.1365-3040.2007.01770.x 18088330

[B75] Perez-Alfocea F Ghanem ME Gomez-Cadenas A Dodd IC Omics of root-to-shoot signaling under salt stress and water deficit Omics 2011 15 12 893 901 10.1089/omi.2011.0092 22136663

[B76] Davies WJ Tardieu F Trejo CL How do chemical signals work in plants that grow in drying soil Plant Physiol 1994 104 2 309 314 12232081 10.1104/pp.104.2.309 PMC159200

[B77] Tardieu F Davies WJ Integration of hydraulic and chemical signalling in the control of stomatal conductance and water status of droughted plants Plant Cell Environ 1993 16 4 341 349 10.1111/j.1365-3040.1993.tb00880.x

[B78] Schachtman DP Goodger JQ Chemical root to shoot signaling under drought Trends Plant Sci 2008 13 6 281 287 10.1016/j.tplants.2008.04.003 18467158

[B79] Davies W Kudoyarova G Hartung W Long-distance ABA Signaling and Its Relation to Other Signaling Pathways in the Detection of Soil Drying and the Mediation of the Plant‚Âôs Response to Drought J Plant Growth Regul 2005 24 4 285 295 10.1007/s00344-005-0103-1

[B80] Tardieu F Parent B Simonneau T Control of leaf growth by abscisic acid: hydraulic or non-hydraulic processes? Plant Cell Environ 2010 33 4 636 647 10.1111/j.1365-3040.2009.02091.x 20002334

[B81] Tang AC Boyer JS Growth-induced water potentials and the growth of maize leaves J Exp Bot 2002 53 368 489 503 10.1093/jexbot/53.368.489 11847248

[B82] Mittler R Vanderauwera S Suzuki N Miller G Tognetti VB Vandepoele K Gollery M Shulaev V Van BF ROS signaling: the new wave? Trends Plant Sci 2011 16 6 300 309 10.1016/j.tplants.2011.03.007 21482172

[B83] Miller G Schlauch K Tam R Cortes D Torres MA Shulaev V Dangl JL Mittler R The plant NADPH oxidase RBOHD mediates rapid systemic signaling in response to diverse stimuli Sci Signal 2009 2 84 ra45 10.1126/scisignal.2000448 19690331

[B84] Manza LL Stamer SL Ham AJ Codreanu SG Liebler DC Sample preparation and digestion for proteomic analyses using spin filters Proteomics 2005 5 7 1742 1745 10.1002/pmic.200401063 15761957

[B85] Wisniewski JR Zougman A Nagaraj N Mann M Universal sample preparation method for proteome analysis Nat Methods 2009 6 5 359 362 10.1038/nmeth.1322 19377485

[B86] Scherl A Shaffer SA Taylor GK Kulasekara HD Miller SI Goodlett DR Genome-specific gas-phase fractionation strategy for improved shotgun proteomic profiling of proteotypic peptides Anal Chem 2008 80 4 1182 1191 10.1021/ac701680f 18211032

[B87] Picardi E Horner DS Chiara M Schiavon R Valle G Pesole G Large-scale detection and analysis of RNA editing in grape mtDNA by RNA deep-sequencing Nucleic Acids Res 2010 38 14 4755 4767 10.1093/nar/gkq202 20385587 PMC2919710

[B88] Goremykin VV Salamini F Velasco R Viola R Mitochondrial DNA of Vitis vinifera and the issue of rampant horizontal gene transfer Mol Biol Evol 2009 26 1 99 110 18922764 10.1093/molbev/msn226

[B89] Zybailov B Mosley AL Sardiu ME Coleman MK Florens L Washburn MP Statistical analysis of membrane proteome expression changes in Saccharomyces cerevisiae J Proteome Res 2006 5 9 2339 2347 10.1021/pr060161n 16944946

[B90] Gammulla CG Pascovici D Atwell BJ Haynes PA Differential metabolic response of cultured rice (Oryza sativa) cells exposed to high- and low-temperature stress Proteomics 2010 10 16 3001 3019 10.1002/pmic.201000054 20645384

[B91] Vizcaino JA Cote R Reisinger F Barsnes H Foster JM Rameseder J Hermjakob H Martens L The Proteomics Identifications database: 2010 update Nucleic Acids Res 2010 38 Database issue D736 D742 19906717 10.1093/nar/gkp964 PMC2808904

